# Protein Kinase C Epsilon Contributes to NADPH Oxidase Activation in a Pre-Eclampsia Lymphoblast Cell Model

**DOI:** 10.3390/diseases1010001

**Published:** 2013-08-28

**Authors:** Toryn M. Poolman, Paulene A. Quinn, Leong Ng

**Affiliations:** Pharmacology and Therapeutics Group, Department of Cardiovascular Sciences, Leicester Royal Infirmary and NIHR Cardiovascular Biomedical Research Unit, Clinical Sciences Building, Leicester, LE2 7LX, UK

**Keywords:** pre-eclampsia, NADPH oxidase, protein kinase C, free radicals, hypertension

## Abstract

Pre-eclampsia is a pregnancy-specific disorder characterised by hypertension and proteinuria, which in severe cases results in multi-system disturbances. The maternal syndrome is associated with a pro-inflammatory state, consisting of leukocyte activation, which is thought to contribute to the widespread endothelial dysfunction. We previously showed increased activation of NADPH oxidase in pre-eclampsia, in both neutrophils and B-lymphoblast cell lines (B-LCLs). In this study, the mechanism by which NADPH oxidase activity is increased in pre-eclampsia was further investigated. NADPH oxidase activity was found to be increased in phorbol-12-myristate-13-acetate (PMA) stimulated B-LCLs isolated from women with pre-eclampsia. This correlated with an increase in protein kinase C (PKC) substrate phosphorylation, p47-phox phosphorylation (a regulatory component of NADPH oxidase) and p47-phox directed-kinase activity. Using ion exchange and hydroxyapatite chromatography we identified a major peak of PMA regulated p47-phox kinase activity. Chromatography fractions were probed for PKC isoforms. We found the major peak of p47-phox kinase activity could not be separated from the elution profile of PKC epsilon. Using a peptide inhibitor of PKC epsilon, PMA-induced reactive oxygen species (ROS) production could be reduced to that of a normal B-LCL. These data suggest a pro-inflammatory role for PKC epsilon in the pathogenesis of pre-eclampsia.

## 1 Introduction

Pre-eclampsia is a pregnancy-specific disorder and a leading cause of maternal morbidity and perinatal death [1]. It is associated with hypertension and proteinuria; however, its origins are early in gestation, where poor implantation and remodelling of the spiral arteries leads to placental underperfusion and hypoxia. This results in the release of placental factors, of which there are many, e.g., soluble vascular endothelial growth factor (VEGF) receptor (sflt) [2]. The maternal syndrome results from a widespread endothelial dysfunction, possibly as a direct consequence of an excessive maternal inflammatory response [3]. The activation of leukocytes, such as neutrophils, is thought to contribute to this inflammatory process through the release of toxic substances, such as reactive oxygen species (ROS) and other microvascular disturbances [4,5].

To provide an appropriate response to a specific stimulus, leukocytes may undergo a priming process that enhances ROS production to agonists, such as tumour necrosis factor (TNF) and granulocyte/macrophage colony-stimulating factor (GM-CSF) [6]. This process is largely controlled by kinases, including phosphatidylinositol-3-kinase (PI3K) and p38MAPK. These kinases have been shown to induce the phosphorylation of the regulatory components of NADPH oxidase, leading to an enhanced oxidative burst [7].

NADPH oxidase consists of a cytosolic trimer of p40-phox, p47-phox and p67-phox, becoming phosphorylated upon activation and then translocating to the cell membrane. A fourth cytosolic subunit, the small guanine binding protein, cytosolic Rac, is found and translocates independently. The membrane component consists of p22-phox and gp91-phox, which bind the cytosolic components upon activation. However, the p47-phox subunit is the most widely accepted regulatory point. This protein is heavily phosphorylated in its *C*-terminal quarter by numerous kinases, such as PKC [8] and Akt [9].

We previously showed in a pre-eclampsia lymphoblast model ROS production was increased in response to phorbol-12-myristate-13-acetate (PMA) [10]. In the current study we show that P47-phox phosphorylation was also increased on PKC phosphorylation sites, correlating with increased ROS production in the pre-eclamptic B-lymphoblast cell lines (B-LCLs). P47-kinase activity was also increased, therefore we determined the kinase responsible for this process. The novel PKC isoform, PKCε, was found to be responsible for this increase and hence highlights an important role for this kinase in the pathogenesis of pre-eclampsia.

## 2 Experimental Section

### 2.1 Materials

Acrylamide (Flowgen Bioscience Ltd., Nottingham, UK), protein G sepharose, β-glycerophosphate, and RO318330 (Merck Biosciences; Nottingham, UK), anti-phospho PKC substrate antibody (Cell Signalling Technologies; Inc., MA, USA), mouse anti-p47-phox antibody and anti-PKC antibodies (BD Biosciences; Belgium), Resource S, Q and heparin columns, horse radish peroxidase (HRP)-coupled rabbit secondary antibody, Hybond-C nitrocellulose membrane and ECL kit (Amersham Biosciences; Little Chalfont, UK), methylacridinium ester (MAE; 4-(2-succinimidyloxycarbonylethyl)phenyl-10-methylacridinium 9-carboxylate fluorosulphonate) (Molecular Light Technology; Cardiff, UK). Microlite 2 microtitre plates were supplied by Dynex Technologies Inc. and hydroxyapatite columns were from Bio-Rad Laboratories; Hemel Hempstead, UK. All other materials were obtained from Sigma (Poole, UK).

### 2.2 Cell Culture

B-lymphoblast cell lines (B-LCLs) (Epstein-Barr virus transformed), isolated and cultured from third-trimester and postpartum pre-eclamptic and normal women, were characterised in a previous study [10]. This study group consisted of 20 cell lines from either normal (NT) or pre-eclamptic (PET) (10 third trimester and postpartum) women.

### 2.3 Measurement of ROS Production

Luminol chemiluminescence (CL) was performed as previously described [11]. ROS production was measured from 2 × 10^5^ cells, stimulated by the addition of 0.001^−1^ μmol/L PMA. The total oxidative capacity was determined from the area under the curve (AUC) and expressed as relative light units (RLU min^−1^). NADPH oxidase activity in cell lysates was measured as previously described [12]. Protein concentration was determined by the fluorescamine assay [13]. ROS production was measured from 50 μg of total lysate in a reaction buffer containing 10 μmol/L lucigenin. ROS production was stimulated with NADPH, GTPγS and SDS. CL was measured continuously for 40 min. NADPH oxidase activity was determined from the AUC and expressed as RLU min^−1^ 50 μg protein content^−1^ [12].

### 2.4 Cell Treatments, Lysis, Immunoprecipitation, Fractionation and SDS-PAGE

B lymphoblasts (3 × 10^6^/mL) were stimulated with PMA (final concentration 1 μM) for the indicated time and lysed on ice by the addition of a triton lysis buffer [11]. Cells were then fractionated by previously described methods [14]. 35 μg of protein was analysed using 10% SDS-polyacrylamide gels and immunoblotting [11] with an anti-phospho PKC substrate, anti-PKCα, anti-PKCβ, anti-PKCδ, anti-PKCε and pan-PKC antibodies. Blots were visualised using a HRP-coupled secondary antibody and an ECL detection system. Hyperfilms were quantified by densitometry using a BioRad densitometer. To test for equal loading, all blots were stripped and probed with anti-tubulin or rabbit anti-p47-phox antibodies. For immunoprecipitation, 500 μg of total cell protein was incubated overnight with 0.5 μg of a mouse anti-p47-phox or PKCε antibody as described in [11].

### 2.5 P47-Phox Kinase Assays

P47-phox and p67-phox were expressed in sf9 cells and purified as previously described using a baculovirus expression system (constructs were a kind gift from JD Lambeth, Emory University, Atlanta, Georgia, GA, USA) [15]. Microlite 2 microtitre plates were coated with p47-phox (0.5 μg per well) in phosphate buffered saline (PBS) overnight. After washing in PBS, the plates were blocked with PBS supplemented with 1% (w/v) bovine serum albumin (BSA) for 1 h. Before use, the plates were washed with PBS. Whole cell lysates were prepared from B lymphoblasts as described above. From each cell lysate a 5 μg/mL solution (diluted in a HEPES-Tween solution (25 mmol/L HEPES, pH 7.3, with 0.01% (v/v) Tween-20) was prepared. 6 μL of this sample was added to each well and the volume made up to 80 μL with HEPES-Tween solution. The plates were then warmed to 37 °C for 10 min and in some experiments either Ro31-8220 (1 μmol/L) or mPKC peptide inhibitor (50 μmol/L) was also included. PMA (1 μmol/L), diacylglycerol analogue (DAG analogue) (OC-DAG (1,2-Dioctanoyl-*sn*-glycerol)) (200 μmol/L), phosphatidylserine (PS) (0.1 mg/mL) and calcium (1 mmol/L) were also included (modified from [16]). Reactions were initiated by the addition of 20 μL of a pre-warmed kinase buffer (final concentration: 20 mmol/L Tris, pH 7.5, 25 mmol/L MgCl_2_, 10 mmol/L β-glycerophosphate, 0.5 mmol/L sodium orthovanadate, 0.5 mmol/L dithiothreitol (DTT) and 1 mmol/L adenosine triphosphate (ATP) and incubated for 30 min at 37 °C. The reaction was terminated by washing the plate in a modified PBS-tween [17]. Phosphorylated p47-phox was detected using an anti-phospho PKC substrate antibody. The anti-phospho PKC substrate antibody is thought to preferentially bind phosphorylated serine residues with an arginine or lysine residue in the −2 and +2 positions and with a hydrophobic residue at the +1 position [18]. Bound antibody was detected using MAE-labelled streptavidin. The CL-signal (expressed as RLU) was detected using a Dynex MLX luminometer [17] Radioactive kinase assays were carried in similar buffer conditions as described above using a [^32^P] ATP (25 μM ATP final concentration).

### 2.6 Fractionation of B-Lymphoblast Cytosol

Cytosol was prepared as previously described [14] and applied to a Resource Q cation-exchange column and washed with 5 column volumes of 5 mmol/L Tris (pH 7.4). The column was eluted with a linear gradient of 0–1 mol/L NaCl. The majority of p47-phox protein kinase activity eluted at 0.2–0.5 mol/L NaCl. Fractions containing PMA-regulated p47-phox kinase activity were subjected to hydroxyapatite chromatography.

### 2.7 Saponin Permeabilization and Inhibition of PKCε

1 × 10^7^ cells were equilibrated at a reduced temperature by two sequential 2-minute incubations, each with 1 mL PBS. The first PBS incubation is carried out at room temperature; the second, with chilled PBS (4 °C). 1.3 mL of a permeabilization buffer (20 mmol/L HEPES, pH 7.4, 10 mmol/L EGTA, 140 mmol/L KCl) was then added. 200 μL aliquots were incubated with the PKCε Translocation Inhibitor Peptide (EAVSLKPT) or a peptide negative control (LSETKPAV) (1 mmol/L). 200 µL of Saponin-permeabilization buffer (20 mmol/L HEPES, pH 7.4, 10 mmol/L EGTA, 140 mmol/L KCl, 12 mmol/L ATP and 100 µg/mL saponin were then added. Cells were further incubated for 10-min on ice and washed four times with 1 mL of chilled PBS. Cells were incubated on ice for 20 min (recovery period), the chilled PBS was removed, 1 mL of room temperature PBS was added and the cells were placed at room temperature for 2 minutes, after which complete cell media was added back to the cells at 37 °C. The cells were further incubated for 30 min at 37 °C before use. The cells were stimulated with 10 nmol/L PMA and ROS production measured as described above.

### 2.8 Data Analysis

All data are presented as mean ± SEM and values were compared using Minitab software (Pennsylvania, PA, USA) with either a Mann-Whitney test or one-way ANOVA, followed by Tukey’s post hoc test. Statistical significance was declared at the *p* < 0.05 level. Areas under the curves (AUC) were calculated using the trapezoidal rule. For Western blotting analysis, all data were normalized to the control group.

## 3 Results and Discussion

### 3.1 ROS Production is Increased in PMA-Stimulated Pre-Eclamptic B-LCLs

**Figure 1:**
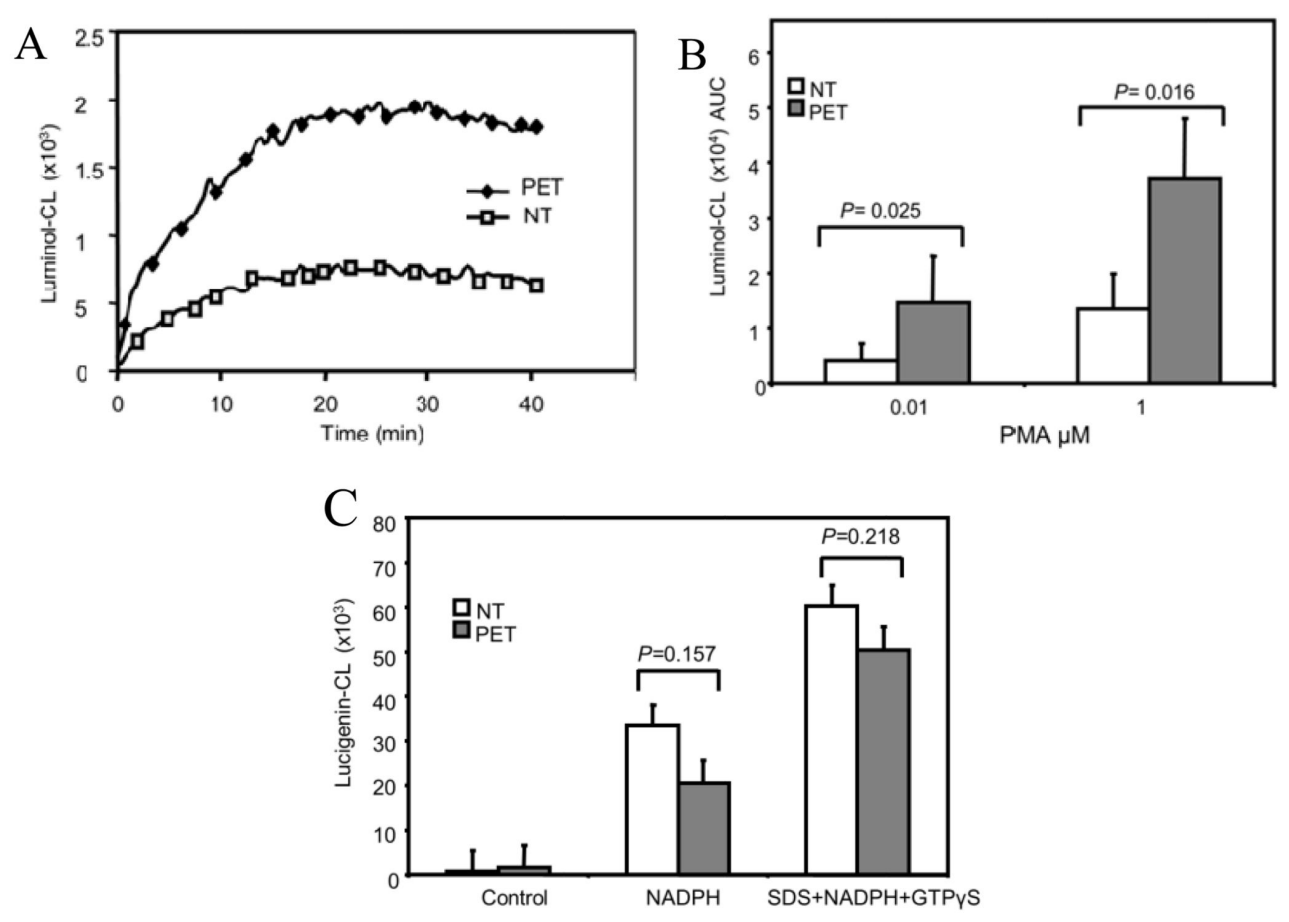
Pre-eclamptic B-lymphoblast cell lines (B-LCLs) display enhanced reactive oxygen species (ROS) production in response to phorbol-12-myristate-13-acetate (PMA). (**A**) Whole cell ROS production was measured from B-LCLs stimulated with 0.01 μmol/L or 1 μmol/L PMA added at time = 0 min and measured by luminol-CL (shown as a representative chemiluminescent (CL) recording). (**B**) The area under the curve (AUC) was calculated for each CL-recording. Each column represents the mean ± SEM for combined results from 10 normal (NT) and 10 pre-eclamptic (PET) B-LCLs stimulated with either PMA dose. (**C**) Cell-free activation in normal and pre-eclamptic B-LCL, cell lysates were stimulated with a combination of NADPH or NADPH with SDS and GTPγS, n = 6 for each cell type. Statistical significance determined using a Mann-Whitney test.

We previously showed that PMA-stimulated ROS production was increased in B-lymphoblast cell lines (B-LCLs) isolated from third trimester and postpartum pre-eclamptic women [10]. The 10 normal and pre-eclamptic B-LCLs (5 third trimester and 5 postpartum) used in this study were all stable cultures displaying increased ROS production in response to PMA, as measured by luminol-CL (Figure 1A,B). ROS production could not be detected in unstimulated cells (not shown). There were no significant differences between pregnancy status and ROS production in either group (*p* > 0.05).

NADPH oxidase activity could also be detected in cell homogenates from B-LCLs using lucigenin-CL (which measures superoxide [O_2_^−^] production). O_2_^−^ production could be measured in cell lysates stimulated with NADPH (with and without SDS and GTPγS). There were no significant differences between normal and pre-eclamptic B-LCLs when NADPH oxidase activity was stimulated in this fashion (Figure 1C).

### 3.2 Increased Kinase Activity and P47-Phox Phosphorylation in B-LCLs

**Figure 2:**
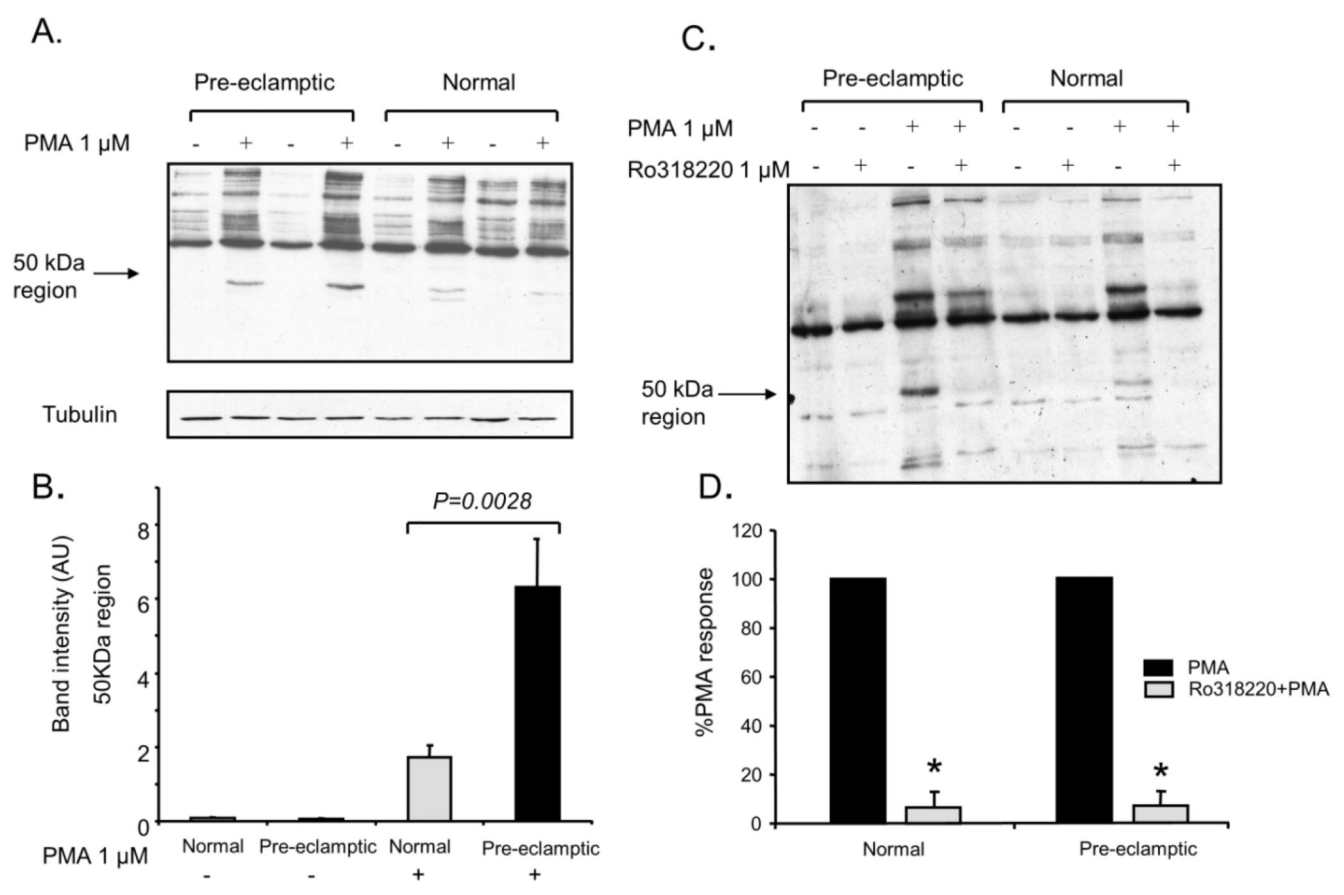
PKC substrate phosphorylation is increased in pre-eclamptic B lymphoblasts. (**A**) Normal and pre-eclamptic B-LCLs were stimulated with 1 μmol/L PMA for 7 min. PKC substrate phosphorylation was determined by western blotting. Pre-eclamptic B-LCLs showed an increase in phosphorylation in a 50 kDa region (*p* = 0.0028) (lower tubulin immunoblots show equal loading). (**B**) Data obtained from immunoblot experiments were analysed using densitometry; each column represents the mean ± SEM from 10 normal and pre-eclamptic B-LCLs. Statistical significance was determined using a Mann-Whitney test (n = 10 for each state). (**C**) Normal and pre-eclamptic B-LCLs were pre-treated with 1 μmol/L Ro31-8220 for 15 min before stimulation with 1 μmol/L PMA. PKC substrate phosphorylation was determined by western blotting. Data obtained from immunoblots experiments were analysed by densitometry and the effect of Ro31-8220 on PKC phosphorylation of a 50 kDa protein was determined (shown as mean ± SEM, n = 3 for each state). Statistical significance from PMA stimulated control cells was determined using a one-way ANOVA (*, *p* > 0.05).

PMA stimulation in normal and pre-eclamptic B-LCLs resulted in the rapid phosphorylation of cellular PKC substrates, as detected using an anti-phospho PKC substrate antibody. Both pre-eclamptic and normal cell lines showed increased phosphorylation on numerous proteins in response to PMA. However, the 60 and 50 kDa regions were the most consistently phosphorylated and easy to identify (Figure 2A). Analysis of the optical density in the 50 kDa region was found to be increased in pre-eclamptic B-LCLs (mean ± SEM; normal 1.7 ± 1.0 *vs.* pre-eclamptic 6.3 ± 4.3 AU, *p* = 0.0028) (Figure 2B). The phosphorylation of the p50 region in response to PMA could be inhibited by pre-treating the cells with 1 μmol/L Ro31-8220 (an inhibitor of protein kinase C [PKC] (Figures 2C and S1). The protein in the 60 kDa region was less affected by Ro31-8220. The sensitivity towards Ro31-8220 did not differ between the two disease states, inhibited by 93.5 ± 6.3% and 93.2 ± 3.5% for normal and pre-eclamptic B-LCLs (mean ± SEM, n = 3) (Figure 2D).

### 3.3 P47-Phox Phosphorylation Is Increased in Pre-Eclamptic B-LCLs

**Figure 3:**
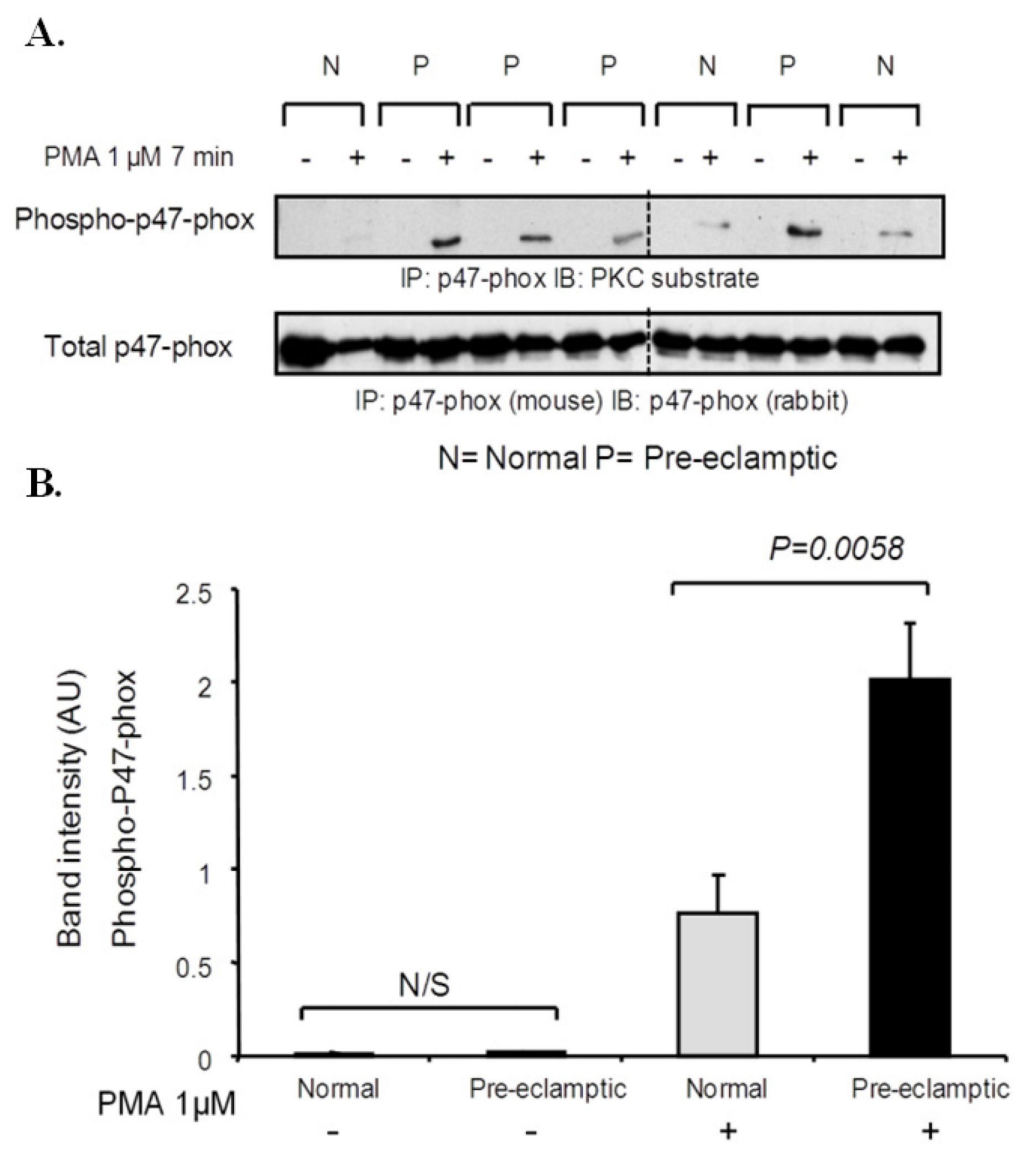
p47-phox phosphorylation is increased in pre-eclamptic B-LCLs. (**A**) Normal and pre-eclamptic B-LCLs were stimulated for 7 minutes with 1 μmol/L PMA. p47-phox was immunoprecipitated from normal and pre-eclamptic B-LCLs (using a mouse anti-p47-phox antibody). Immunoblots were then probed for PKC substrate phosphorylation. The identity of p47-phox and equal loading was determined using a rabbit anti-p47-phox antibody. (**B**) Data obtained from immunoblot experiments were analysed by densitometry, each column represents the mean ± SEM for 10 normal and pre-eclamptic B-LCLs. Pre-eclamptic B-LCLs showed an increase in p47-phox phosphorylation (*p* = 0.0058). Statistical significance was determined using a Mann-Whitney test. Dashed line indicates separate blots (see Figure S3 for full image).

P47-phox immunoprecipitates were prepared from PMA stimulated normal and pre-eclamptic B-LCLs. Immunoblotting with an anti-phospho PKC substrate antibody revealed that p47-phox phosphorylation was increased in pre-eclamptic B-LCLs (mean ± SEM; normal 0.8 ± 0.6 *vs.* pre-eclamptic 2.0 ± 0.9, *p* = 0.0058, n = 10) (Figures 3A,B and S3). The basal level of p47-phox phosphorylation was found to be very low and did not differ between the two cell types (*p* > 0.05).

### 3.4 PKC Expression Is Unchanged between Normal and Pre-Eclamptic B-LCLs

PMA has been shown to stimulate ROS production in B-LCLs and is inhibited by the PKC inhibitor Ro31-8220 [10]. The expression of PKC isoforms were determined by western blotting. The expression of PKCα, PKCβ, PKCδ and PKCε could be measured in these B lymphoblasts, whereas PKCθ and PKCλ were found to be much less. The expression of PKC isoforms α, β, δ and ε (normal *vs.* pre-eclamptic; mean ± SEM) was found to be similar (PKCα, 3.6 ± 2.6 *vs.* 5.8 ± 3.8; PKCβ, 2.3 ± 0.7 *vs.* 2.1 ± 1.4; PKCδ, 3.6 ± 1.8 *vs.* 2.8 ± 1.1; and PKCε, 2.5 ± 1.3 *vs.* 2.7 ± 1.8 normalized densitometric arbitrary units). The protein expression of PKCλ and PKCθ isoforms was at a much lower level than the other PKC isoforms, with no statistical differences between the normal and pre-eclamptic groups (See Figure S2).

### 3.5 P47-Phox Kinase Activity Is Increased in Pre-Eclamptic B-LCLs

**Figure 4:**
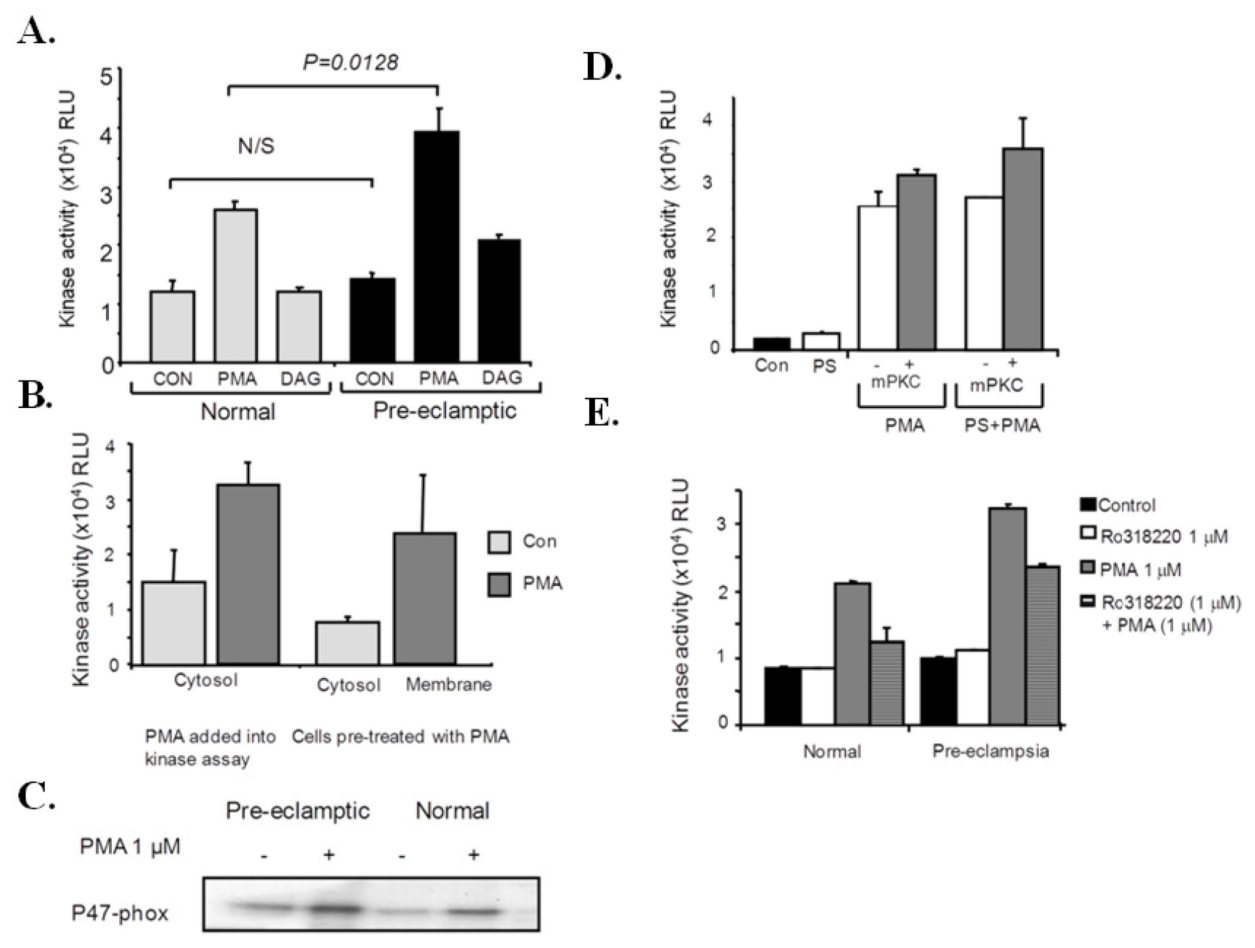
p47-phox kinase activity is increased in pre-eclamptic B-LCLs. (**A**) Cell lysates were prepared from normal and pre-eclamptic B-LCLs. p47-phox kinase activity was determined using a non-radioactive kinase assay. Phosphorylated p47-phox was determined using a PKC substrate antibody. Kinase activities from normal and pre-eclamptic B-LCLs are expressed as RLU, with each column representing the mean kinase activity (±SEM). Pre-eclamptic B-LCLs display an increase in PMA-induced p47-phox kinase activity (*p* = 0.0128). Statistical significance was determined using a Mann-Whitney test (n = 10 for each state). The effect of a DAG analogue on p47-phox kinase activity was also tested (shown as mean RLU ± SEM, n = 3). (**B**) p47-phox kinase activity was determined in membrane and cytosol fractions from pre-eclamptic B lymphoblasts (stimulated with PMA before (cells pre-treated with PMA) and after fractionation (PMA added into the kinase assay)), each column represents the mean (±SEM) kinase activity for 3 pre-eclamptic B-LCLs. (**C**) PMA-induced kinase activity was determined in cytosol prepared from normal and pre-eclamptic B lymphoblasts and measured using a radioactive kinase assay (n = 3), kinase activity was determined using recombinant p47-phox and [^32^P] ATP. An autoradiogram for phosphorylated p47-phox is shown. (**D**) The effect of a mPKC peptide inhibitor (50 μmol/L) and phosphatidylserine (PS) 0.1 mg/ml on p47-phox activity was measured in whole cell lysates; each column represents the mean kinase activity (RLU ± SEM) for 3 pre-eclamptic B-LCLs. (**E**) The effect of Ro31-8220 (1 μmol/L) on PMA-induced p47-phox kinase activity measured in whole cell lysates; each column represents the mean kinase activity (RLU ± SEM) for 3 pre-eclamptic B-LCLs. Statistical significance from PMA stimulated control lysates was determined using a one-way ANOVA (*, *p* < 0.05).

To determine if p47-phox kinase activity was increased in pre-eclamptic B-LCLs a non-radioactive kinase assay was developed to measure p47-phox kinase activity in whole cell lysates. In resting cells a basal p47-phox kinase activity could be measured, which could be rapidly enhanced by the addition of PMA directly into the kinase assay. The basal p47-phox kinase activity was not found to be significantly different between the two cell types (normal *vs.* pre-eclamptic, mean ± SEM; 10.1 ± 4.9 *vs.* 14.1 ± 3.5 × 10^3^ RLU, n = 10). Pre-eclamptic B-LCLs were found to have increased kinase activity in response to PMA, when PMA was added directly into the kinase assay (normal *vs.* pre-eclamptic, mean ± SEM; 24.8 ± 11 *vs.* 39.2 ± 12.8 × 10^3^ RLU, *p* = 0.0128, n = 10) (Figure 4A). A DAG analogue was also able to induce p47-phox phosphorylation, albeit to a lesser extent to that found with PMA (Figure 4A). p67-phox kinase activity could not be detected in cell lysates using the phospho-PKC substrate antibody (not shown). The PMA-sensitive kinase was found in the cytosol of resting cells, whereas membrane fractions contained the most activity if cells were stimulated with PMA prior to cell fractionation (Figure 4B). A radioactive kinase assay was also used to confirm the presence of a PMA-sensitive kinase found in B-LCL cytosol (Figure 4C).

A peptide inhibitor of PKCα and β (mPKC inhibitor) did not inhibit PMA-induced p47-kinase activity (Figure 4D). If the PKC cofactor PS was added into the assay, kinase activity was not increased. Kinase activity was also found to be calcium independent (not shown) and all subsequent reactions were carried out in the presence of EGTA. PMA-induced p47-phox kinase activity was inhibited by the non-specific PKC inhibitor, Ro31-8220 (using 1 mmol/L ATP) (Figure 4E).

### 3.6 Analysis of P47-Phox Kinase Activity in Pre-Eclamptic B-LCLs

**Figure 5:**
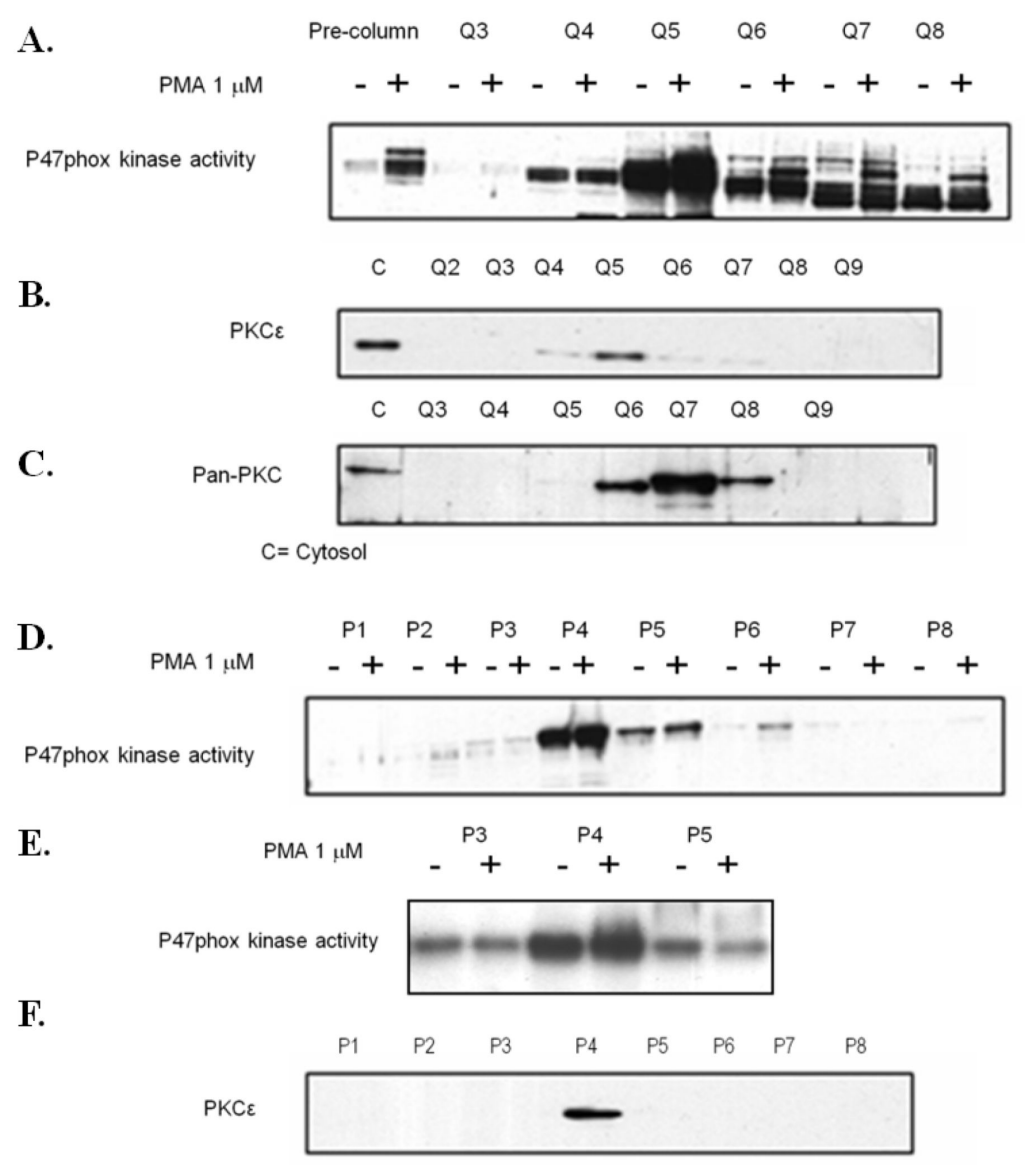
Identification of a major p47-phox kinase in pre-eclamptic B-LCLs. Cytosolic preparations were fractionated by ion exchange chromatography (using a Resource Q column as described in the material and methods), kinase activity (non-radioactive kinase assay) (**A**) and the expression profile of PKC isoforms (**B** and **C**) was determined for each fraction. Fractions 4 and 5 from the ion exchange column were subjected to fractionation using a hydroxyapatite column, with kinase activity (non-radioactive **D** and **E** using [^32^P] ATP) and the presence of PKCε (**F**) determined for each fraction.

The apparent increase in kinase activity in the pre-eclamptic cell lines was further investigated. Cytosol was applied to a Q-column and fractionated. Kinase activity and PKC levels in each fraction were then tested. The major peak of activity was strongly associated with the presence of PKCε. Using a pan-PKC antibody (for PKCα, βI, βII and γ) the remaining peak of activity could be determined (Figure 5A–C). The major peak of activity was further purified on a hydroxyapatite column, where the peak of activity was found to correlate with the presence of PKCε (Figure 5D–F).

### 3.7 PKCε Activation in Pre-Eclamptic B-LCLs

**Figure 6:**
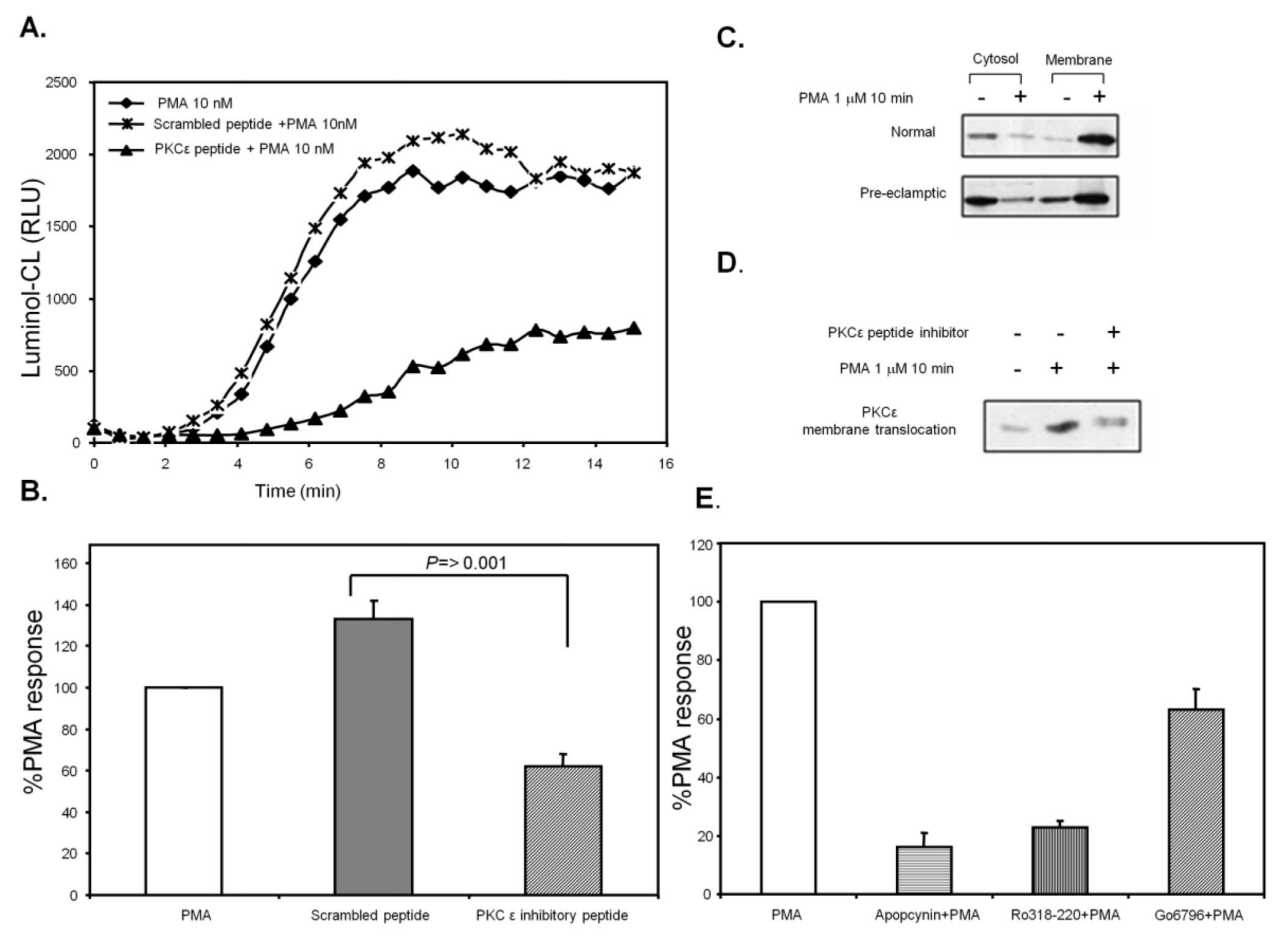
The effect of PKCε inhibition on ROS production in pre-eclamptic B-LCLs. (**A**) Pre-eclamptic B-LCLs were permeabilized with saponin and incubated with either a PKCε translocation inhibitor peptide (EAVSLKPT) or a scrambled negative control peptide (LSETKPAV). Whole cell ROS production was measured from B-LCLs stimulated with 0.01 μmol/L PMA added at time = 0 minutes and measured by luminol-CL (shown as a representative chemiluminescent (CL) recording). (**B**) The AUC was calculated for each CL-recording and represented as %PMA response (mean ± SEM) for combined results from 6 pre-eclamptic B-LCLs. Statistical significance was determined by a One-way ANOVA. (**C**) Membrane translocation of PKCε in normal and pre-eclamptic B-LCLs. (**D**) The effect of the PKCε translocation inhibitor peptide on PMA-induced translocation of PKCε. (**E**) Pre-eclamptic B-LCLs were treated with either apocynin, Ro318220 or Go6796 before being stimulated with 1 μmol/L PMA, here represented as %PMA response.

B-LCLs proved to be very difficult to transfect, making the use of gene knockdown methods such as siRNA a challenge. The most appropriate method to reduce the activation of PKCε in the cells was to use an inhibitor of PKCε translocation, previously shown to be effective and delivered in to the cell by a transient permeablization method (saponin). Using this peptide (and a scrambled control peptide), ROS production was found to be reduced (by greater than 50%) (Figure 6A,B). We were unable to measure NADPH oxidase activity in normal (NT) cells by this method, possibly due to the low levels of enzyme activity in this group. PKCε translocation was also found to be unchanged between the two cell types (Figure 6C). The basal level of PKCε association was also found not to be significantly different in the two cell types. The translocation inhibitor was found to effectively inhibit membrane translocation of PKCε (Figure 6D). ROS production in these cells was found to be inhibited by apocynin (a general inhibitor of NADPH oxidase) and as expected the PKC inhibitors Ro318220 and Go6796. Interestingly, there was a difference between the two PKC inhibitors (Figure 6E). Go6796 has been previously shown to be ineffective against PKCε [19].

Pre-eclampsia is associated with leukocyte activation [3] which is thought to contribute towards the disease process through numerous mechanisms, including the activation of NADPH oxidase and subsequent ROS production. Oxidative stress and a pro-inflammatory state are features of the maternal syndrome. However, leukocyte activation and entrapment in the microcirculation may affect whole organ perfusion pressure, leading to organ damage [20,21]. Neutrophils isolated from women with pre-eclampsia have been found to be hyper-responsive to agonists, such as formyl-methionyl-leucyl-phenylalanine (fMLP) [4,22,23] and PMA [4]. B-LCLs isolated from women with pre-eclampsia also show this difference in response to PMA. Interestingly, this phenomenon was still evident 6 months after parturition. Our previous work has suggested that these differences were not due to any changes in the expression levels of the subunits of NADPH oxidase [10] and in this study we show data that implicates the activation of PKCε.

The role of PKC in vascular disease has been well established. PKCε has been shown to play an important role in the pathogenesis of cardiovascular disease (reviewed in [24]). In particular, the progression of cardiac hypertrophy, failure [25], and fibrosis [26]. Interestingly, pre-eclampsia has been shown to be associated with multiple postpartum cardiovascular impairments, such as left ventricular dysfunction and hypertension [27]. However, to date the only association of PKCε in pre-eclampsia has been demonstrated with endothelial cells that treated with serum from pre-eclamptic women, showing an increase in membrane association of PKCε. Therefore, increased PKCε activation has the potential to contribute to the long term cardiovascular disturbances seen in pre-eclampsia.

Firstly, the increased responsiveness to PMA was not due to a detectable change in PKC isoform expression. However, neutrophils isolated from diabetic patients showed an increase in ROS production in response to PMA and which correlated with the increased expression of PKC [28]. Neutrophils isolated from patients with rheumatoid arthritis have been shown to be hyper-responsive to PMA [29] and phosphorylation on serine 345 mediates the priming effect [30]. Secondly, we found that phosphorylation of PKC substrates revealed that a protein in a region of 50 kDa was significantly increased. Moreover, p47-phox phosphorylation and kinase activity also was increased in PMA-stimulated B-LCLs isolated from pre-eclamptic subjects. These results suggested that the pre-eclamptic phenotype was associated with an increase in PKC activity or an associated kinase.

Finally, we showed that kinase activity was increased in B-LCLs isolated from pre-eclamptic subjects. Cell fractionation experiments revealed a kinase located in the cytosol of B lymphoblasts, which could be directly stimulated by adding PMA into the kinase assay. Translocation of this kinase to membrane fractions was demonstrated by PMA stimulation of cells. The activity of this kinase was also found to be calcium-independent. Cytosolic fractions from pre-eclamptic B-LCLs were subjected to ion exchange and hydroxyapatite chromatography. This suggested that the major peak of kinase activity in these cells was associated with the presence of PKCε.

The importance of p47-phox phosphorylation has been previously demonstrated in B-LCLs [31–33]. The major sites of phosphorylation in the NADPH oxidase complex are serine residues in the *C-*terminal quarter of p47-phox (9 serines between residues 303 and 379 [31]). We used an anti-phospho-(ser) PKC substrate antibody (detecting phosphorylated serines surround by a basic residue at 2−/2+ and a hydrophobic residue at +1) to detect enhanced PKC activity and p47-phox phosphorylation in cells from pre-eclamptic subjects. However, the basal level of p47-phox phosphorylation in B-LCLs isolated from pre-eclamptic or normal subjects was very low or on different phosphorylation sites to those detected by the antibody. This antibody theoretically detects phosphorylation on a subset of functionally important serine residues, serine 303, 304 and 328 of p47-phox [18], but may also detect other phosphorylated serine residues with a similar motif. Serines 303 and 304 are heavily phosphorylated after cell activation and are both required for oxidase activity, while serine 328 has been shown to be a major phosphorylation site for PKC isoforms [34]. Phosphorylation of p47-phox initiates the activation of NADPH oxidase, allowing the translocation of the cytosolic subunits to the cell membrane. Upon cell stimulation, cytosolic p47-phox is phosphorylated on serine 359 and/or 370. Following membrane translocation of partially phosphorylated p47-phox, serines 303 and/or 304 are then phosphorylated [33].

Novel PKC isoforms have been previously shown to be critical in the activation of NADPH oxidase in monocytes [35]. However, the direct activation of NADPH oxidase by PKCε has not been extensively studied. PKCε has a similar substrate specificity to other PKC isoforms; however, it shows less specificity with only a strong requirement for a basic/charged residue at −3 and −2 positions from the target serine[36]. However, kinase substrate specificity can be influenced by many additional interactions *in vitro.*

We used a peptide inhibitor of PKCε, which inhibits the membrane translocation of the enzyme rather than activity per se [37], its specificity has been widely demonstrated [38]. This inhibitor was able to reduce ROS production in the pre-eclamptic cells by 50%, which may account for the priming mechanism in the pre-eclamptic cells. ROS production was completely inhibited by Ro318220 and to a much lesser extent Go6796. Go6796 is not an effective inhibitor of PKCε [19]; however, caution should be taken when analysing data regarding the specificity of these compounds. PKCε activation has been implemented in multiple cardiovascular disorders [39,40], where interestingly its activation has also been associated with a protective role. The mechanism by which PKCε activity is increased in pre-eclamptic B-LCLs is unclear. The levels of PKCε and its membrane translocation did not reach significance in pre-eclamptic B-LCLs; however, this could contribute to the priming mechanism for increase ROS production. Similar mechanisms have been demonstrated in neutrophils [30]. Further investigation into the regulation of PKCε in B-LCLs is warranted. Human umbilical endothelial cells that are treated with serum from pre-eclamptic women showed that there was an increase in membrane association of PKCε [41], which further indicates a possible role for this kinase. PKCε is essential for VEGF-stimulated phosphorylation of Akt, eNOS and the catalytic activity of NO synthase in endothelial cells [42], factors that are also important in pre-eclampsia [5].

## 4 Conclusions

These data indicate that PKCε is responsible for the enhanced NADPH oxidase activity associated with leukocytes in pre-eclampsia. Leukocyte activation and the subsequent ROS production (from NADPH oxidase) are thought to be a contributory factor in the underlying endothelial dysfunction associated with pre-eclampsia. Further investigations to elucidate the nature of PKCε may reveal future therapeutic targets that may limit leukocyte activation and the production of free radicals from leukocytes in pre-eclampsia.

## Supplementary Materials

Supplementary materials can be accessed at: http://www.mdpi.com/2079-9721/1/1/1/s1.

Supplementary Materials

## References

[1] Roberts JM, Gammill HS (2005). Preeclampsia: Recent insights. Hypertension.

[2] Maynard SE, Min JY, Merchan J, Lim KH, Li J, Mondal S, Libermann TA, Morgan JP, Sellke FW, Stillman IE (2003). Excess placental soluble fms-like tyrosine kinase 1 (sflt1) may contribute to endothelial dysfunction, Hypertension, and proteinuria in preeclampsia. J Clin Invest.

[3] Redman CW, Sacks GP, Sargent IL (1999). Preeclampsia: An excessive maternal inflammatory response to pregnancy. Am J Obstet Gynecol.

[4] Lee VM, Quinn PA, Jennings SC, Ng LL (2003). Neutrophil activation and production of reactive oxygen species in pre-eclampsia. J Hypertens.

[5] Young BC, Levine RJ, Karumanchi SA (2010). Pathogenesis of preeclampsia. Ann Rev Pathol.

[6] Dang PM, Dewas C, Gaudry M, Fay M, Pedruzzi E, Gougerot-Pocidalo MA, El Benna J (1999). Priming of human neutrophil respiratory burst by granulocyte/macrophage colony-stimulating factor (gm-csf) involves partial phosphorylation of p47-phox. J Biol Chem.

[7] Dang PM, Morel F, Gougerot-Pocidalo MA, Benna JE (2003). Phosphorylation of the nadph oxidase component p67-phox by erk2 and p38mapk: Selectivity of phosphorylated sites and existence of an intramolecular regulatory domain in the tetratricopeptide-rich region. Biochemistry.

[8] Reeves EP, Dekker LV, Forbes LV, Wientjes FB, Grogan A, Pappin DJ, Segal AW (1999). Direct interaction between p47phox and protein kinase C: Evidence for targeting of protein kinase C by p47phox in neutrophils. Biochem J.

[9] Hoyal CR, Gutierrez A, Young BM, Catz SD, Lin JH, Tsichlis PN, Babior BM (2003). Modulation of p47phox activity by site-specific phosphorylation: Akt-dependent activation of the nadph oxidase. Proc Natl Acad Sci USA.

[10] Lee VM, Quinn PA, Jennings SC, Ng LL (2003). Nadph oxidase activity in preeclampsia with immortalized lymphoblasts used as models. Hypertension.

[11] Poolman TM, Ng LL, Farmer PB, Manson MM (2005). Inhibition of the respiratory burst by resveratrol in human monocytes: Correlation with inhibition of pi3k signaling. Free Radic Biol Med.

[12] Kitada M, Koya D, Sugimoto T, Isono M, Araki S, Kashiwagi A, Haneda M (2003). Translocation of glomerular p47phox and p67phox by protein kinase C-beta activation is required for oxidative stress in diabetic nephropathy. Diabetes.

[13] Bohlen P, Stein S, Dairman W, Udenfriend S (1973). Fluorometric assay of proteins in the nanogram range. Arch Biochem Biophys.

[14] Cachia O, Benna JE, Pedruzzi E, Descomps B, Gougerot-Pocidalo MA, Leger CL (1998). Alpha-tocopherol inhibits the respiratory burst in human monocytes. Attenuation of p47-phox membrane translocation and phosphorylation. J Biol Chem.

[15] Uhlinger DJ, Inge KL, Kreck ML, Tyagi SR, Neckelmann N, Lambeth JD (1992). Reconstitution and characterization of the human neutrophil respiratory burst oxidase using recombinant p47-phox, p67-phox and plasma membrane. Biochem Biophys Res Commun.

[16] Palmer RH, Dekker LV, Woscholski R, Le Good JA, Gigg R, Parker PJ (1995). Activation of prk1 by phosphatidylinositol 4,5-bisphosphate and phosphatidylinositol 3,4,5-trisphosphate. J Biol Chem.

[17] Ng LL, O'Brien RJ, Demme B, Jennings S (2002). Non-competitive immunochemiluminometric assay for cardiotrophin-1 detects elevated plasma levels in human heart failure. Clin Sci.

[18] Ohira T, Zhan Q, Ge Q, VanDyke T, Badwey JA (2003). Protein phosphorylation in neutrophils monitored with phosphospecific antibodies. J Immunol Methods.

[19] Martiny-Baron G, Kazanietz MG, Mischak H, Blumberg PM, Kochs G, Hug H, Marme D, Schachtele C (1993). Selective inhibition of protein kinase C isozymes by the indolocarbazole go 6976. J Biol Chem.

[20] Mellembakken JR, Aukrust P, Olafsen MK, Ueland T, Hestdal K, Videm V (2002). Activation of leukocytes during the uteroplacental passage in preeclampsia. Hypertension.

[21] Schmid-Schonbein GW (1993). The damaging potential of leukocyte activation in the microcirculation. Angiology.

[22] Tsukimori K, Maeda H, Ishida K, Nagata H, Koyanagi T, Nakano H (1993). The superoxide generation of neutrophils in normal and preeclamptic pregnancies. Obstet Gynecol.

[23] Tsukimori K, Fukushima K, Tsushima A, Nakano H (2005). Generation of reactive oxygen species by neutrophils and endothelial cell injury in normal and preeclamptic pregnancies. Hypertension.

[24] Ferreira JC, Brum PC, Mochly-Rosen D (2011). Biipkc and εpkc isozymes as potential pharmacological targets in cardiac hypertrophy and heart failure. J Mol Cell Cardiol.

[25] Takeishi Y, Ping P, Bolli R, Kirkpatrick DL, Hoit BD, Walsh RA (2000). Transgenic overexpression of constitutively active protein kinase C epsilon causes concentric cardiac hypertrophy. Circ Res.

[26] Inagaki K, Koyanagi T, Berry NC, Sun L, Mochly-Rosen D (2008). Pharmacological inhibition of epsilon-protein kinase C attenuates cardiac fibrosis and dysfunction in hypertension-induced heart failure. Hypertension.

[27] Melchiorre K, Sutherland GR, Liberati M, Thilaganathan B (2011). Preeclampsia is associated with persistent postpartum cardiovascular impairment. Hypertension.

[28] Karima M, Kantarci A, Ohira T, Hasturk H, Jones VL, Nam B-H, Malabanan A, Trackman PC, Badwey JA, van Dyke TE (2005). Enhanced superoxide release and elevated protein kinase C activity in neutrophils from diabetic patients: Association with periodontitis. J Leukocyte Biol.

[29] El Benna J, Hayem G, Dang PM, Fay M, Chollet-Martin S, Elbim C, Meyer O, Gougerot-Pocidalo MA (2002). Nadph oxidase priming and p47phox phosphorylation in neutrophils from synovial fluid of patients with rheumatoid arthritis and spondylarthropathy. Inflammation.

[30] Dang PM, Stensballe A, Boussetta T, Raad H, Dewas C, Kroviarski Y, Hayem G, Jensen ON, Gougerot-Pocidalo MA, El-Benna J (2006). A specific p47phox -serine phosphorylated by convergent mapks mediates neutrophil nadph oxidase priming at inflammatory sites. J Clin Invest.

[31] Faust LR, el Benna J, Babior BM, Chanock SJ (1995). The phosphorylation targets of p47phox, a subunit of the respiratory burst oxidase. Functions of the individual target serines as evaluated by site-directed mutagenesis. J Clin Invest.

[32] Inanami O, Johnson JL, McAdara JK, Benna JE, Faust LR, Newburger PE, Babior BM (1998). Activation of the leukocyte nadph oxidase by phorbol ester requires the phosphorylation of p47phox on serine 303 or 304. J Biol Chem.

[33] Johnson JL, Park JW, Benna JE, Faust LP, Inanami O, Babior BM (1998). Activation of p47-phox, a cytosolic subunit of the leukocyte nadph oxidase. Phosphorylation of ser-359 or ser-370 precedes phosphorylation at other sites and is required for activity. J Biol Chem.

[34] Fontayne A, Dang PM, Gougerot-Pocidalo MA, El-Benna J (2002). Phosphorylation of p47phox sites by pkc alpha, beta ii, delta, and zeta: Effect on binding to p22phox and on nadph oxidase activation. Biochemistry.

[35] Bey EA, Xu B, Bhattacharjee A, Oldfield CM, Zhao X, Li Q, Subbulakshmi V, Feldman GM, Wientjes FB, Cathcart MK (2004). Protein kinase C delta is required for p47phox phosphorylation and translocation in activated human monocytes. J Immunol.

[36] Nishikawa K, Toker A, Johannes FJ, Songyang Z, Cantley LC (1997). Determination of the specific substrate sequence motifs of protein kinase C isozymes. J Biol Chem.

[37] Johnson JA, Gray MO, Chen CH, Mochly-Rosen D (1996). A protein kinase C translocation inhibitor as an isozyme-selective antagonist of cardiac function. J Biol Chem.

[38] Palaniyandi SS, Inagaki K, Mochly-Rosen D (2008). Mast cells and epsilonpkc: A role in cardiac remodeling in hypertension-induced heart failure. J Mol Cell Cardiol.

[39] Murriel CL, Mochly-Rosen D (2003). Opposing roles of [delta] and [var epsilon]pkc in cardiac ischemia and reperfusion: Targeting the apoptotic machinery. Arch Biochem Biophys.

[40] Ping P, Song C, Zhang J, Guo Y, Cao X, Li RC, Wu W, Vondriska TM, Pass JM, Tang XL (2002). Formation of protein kinase C(epsilon)-lck signaling modules confers cardioprotection. J Clin Invest.

[41] Haller H, Hempel A, Homuth V, Mandelkow A, Busjahn A, Maasch C, Drab M, Lindschau C, Jupner A, Vetter K (1998). Endothelial-cell permeability and protein kinase C in pre-eclampsia. The Lancet.

[42] Rask-Madsen C, King GL (2008). Differential regulation of vegf signaling by pkc-alpha and pkc-epsilon in endothelial cells. Arterioscler Thromb Vasc Biol.

