# Supplementary material for: Protein Kinase C Epsilon Contributes to NADPH Oxidase Activation in a Pre-Eclampsia Lymphoblast Cell Model
Source: Diseases. Author manuscript; Available in PMC 2016 Nov 25. (PMC5123718; doi:10.3390/diseases1010001)

Supplementary Materials

Figure S1. Loading controls for Figure 2C. Normal and pre-eclamptic B-LCLs were pre-treated with 1 μmol/L Ro31-8220 for 15 minutes before stimulation with 1 μmol/L PMA. Tubulin levels were determined by western blotting.

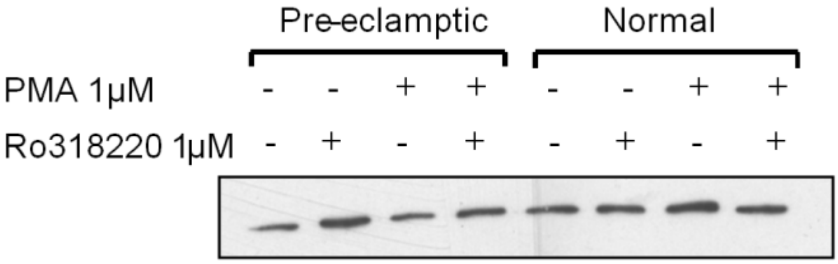

Figure S2. PKC protein levels are unchanged in B-LCLs isolated from pre-eclamptic (P) and normal (N) subjects. (**A**) PKC protein expression levels were determined in normal and pre-eclamptic cell extracts as determined by Western blotting. (**B**) Subsequent immunoblots were analysed by using densitometry, each column represents the mean ± SEM for 6 B-LCLs isolated from normal and pre-eclamptic subjects (results shown for PKCα, PKCβ, PKCδ and PKCε). Statistical significance was determined using a Mann-Whitney test (N/S, n = 6 for each state).

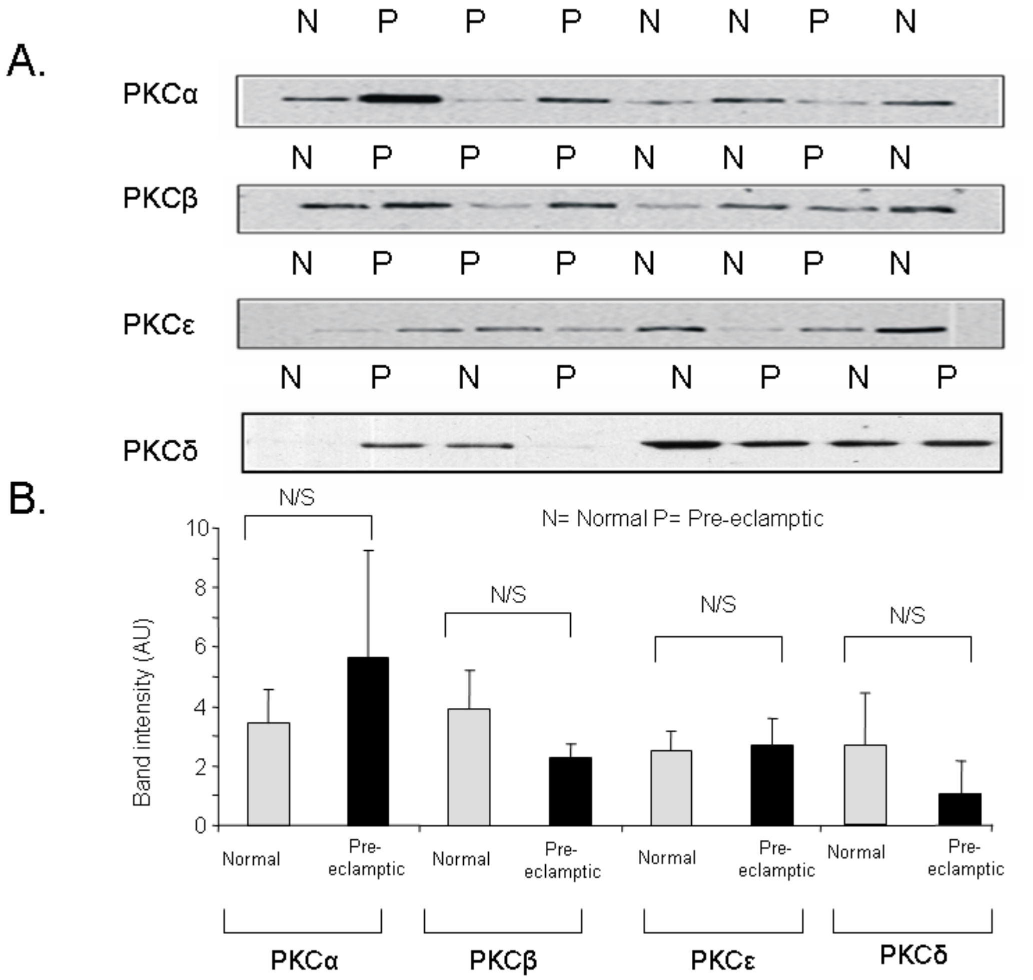

Figure S3. Full gel image for Figure 3A. p47-phox phosphorylation is increased in pre-eclamptic B-LCLs. Normal and pre-eclamptic B-LCLs were stimulated for 7 minutes with 1 μmol/L PMA. p47-phox was immunoprecipitated from normal and pre-eclamptic B-LCLs (using a mouse anti-p47-phox antibody). Immunoblots were then probed for PKC substrate phosphorylation. The identity of p47-phox and equal loading was determined using a rabbit anti-p47-phox antibody.

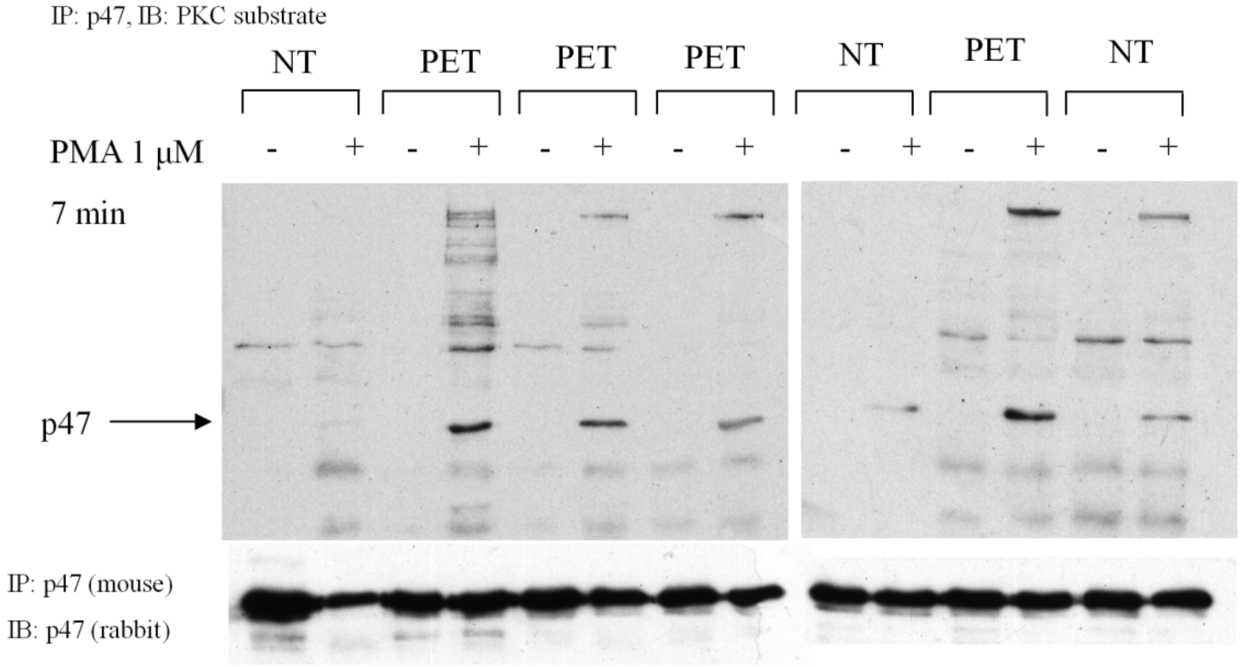

Supplement: Supplementary Materials [file NIHMS70449-supplement-Supplementary_Materials.pdf]
